# Ecological Niche Model of *Bacillus cereus* Group Isolates Containing a Homologue of the pXO1 Anthrax Toxin Genes Infecting Metalworkers in the United States

**DOI:** 10.3390/pathogens11040470

**Published:** 2022-04-14

**Authors:** Mark A. Deka, Chung K. Marston, Julia Garcia-Diaz, Rahsaan Drumgoole, Rita M. Traxler

**Affiliations:** 1 Centers for Disease Control and Prevention, Atlanta, GA 30329, USA; cdk5@cdc.gov; 2 Department of Infectious Disease, Ochsner Medical Center, New Orleans, LA 70121, USA; jgarcia-diaz@ochsner.org; 3 Texas Department of State Health Services, Austin, TX 78756, USA; rahsaan.drumgoole@dshs.texas.gov

**Keywords:** *Bacillus cereus* group, pXO1, welder anthrax, ecological niche modeling

## Abstract

While *Bacillus cereus* typically causes opportunistic infections in humans, within the last three decades, severe and fatal infections caused by isolates of the *B. cereus* group harboring anthrax toxin genes have been reported in the United States. From 1994 to 2020, seven cases of anthrax-like illness resulting from these isolates have been identified. With one exception, the cases have occurred in the Gulf States region of the United States among metalworkers. We aimed to develop an ecological niche model (ENM) to estimate a spatial area conducive to the survival of these organisms based on the presence of known human infections and environmental variables. The estimated ecological niche for *B. cereus* was modeled with the maximum entropy algorithm (Maxent). Environmental variables contributing most to the model were soil characteristics (cation exchange capacity, carbon content, soil pH), temperature, enhanced vegetation index (EVI), and land surface temperature (LST). Much of the suitable environments were located throughout the Gulf Coast Plain, Texas Backland Prairies, East Central Texas Plains, Edwards Plateau, Cross Timbers, Mississippi Alluvial Plain, and Central Great Plains. These findings may provide additional guidance to narrow potential risk areas to efficiently communicate messages to metalworkers and potentially identify individuals who may benefit from the anthrax vaccine.

## 1. Introduction

Severe and fatal infections due to the *Bacillus cereus* group containing a homologue of the pXO1 anthrax toxin genes have been reported in the Gulf states of the United States since the 1990s [1,2,3,4,5]. As of 2021, eight infections have been reported in the literature or by the U.S. Centers for Disease Control and Prevention (CDC) [1,2,3,4,5,6], although how many infections have been missed is unknown. These *B. cereus* group isolates are closely related genetically by multiple-locus sequence typing (MLST) and/or whole-genome sequencing (WGS) [2] and are currently classified as either *B. cereus* or *B. tropicus* based on updated *B. cereus* group taxonomy [7]. These infections appear to be roughly correlated spatially and occupationally. All but one of the identified naturally acquired infections have occurred in Texas and Louisiana among metalworkers or welders, with the remaining infection (a cutaneous case) occurring in a patient who resided in Florida with no metalworking history [5]. Another cutaneous infection occurred due to occupational exposure to a known isolate, G9241, in a research laboratory in Illinois; neither cutaneous case is included in the primary analysis [8].

Information on specific materials or types of welding performed is not available for most patients. Environmental sampling resulting from a public health investigation in the most recent Louisiana case showed nearly identical strains recovered from the patient and the patient’s work environment [6]. Another Texas case investigation recovered a *B. cereus* strain from the worksite of a welder with a fatal infection; however, the strains did not match that of the patient [3]. Environmental sampling has either not been attempted or not been successful at recovering *B. cereus* strains from the work or home environments of other patients with these *B.* *cereus* group infections. These cases are primarily linked by occupation and geographic region, although the rarity of these infections indicates that host factors must have a role in disease development [9]. 

Ecological niche models (ENM) are widely used to generate risk maps in spatial epidemiology to answer questions related to complex disease systems [10,11]. For example, there is a large body of evidence supporting an ecological niche for *B. anthracis*. At the same time, there is evidence that *Bacillus cereus* biovar *anthracis* may have a divergent niche with some geographic overlap with *B. anthracis*, all members of the *B. cereus* group [12,13]. In addition to these known ecological associations of this group of organisms, and although there are few known infections, there appears to be a spatial association among these anthrax-like *B. cereus* infections for which an ENM could provide information that may help narrow the potential areas of risk, which can limit the scope and increase the meaningfulness of risk communication messages to metalworkers. Thus, we aimed to develop an ecological niche model to estimate a spatial area conducive to the survival of these organisms based on the presence of known human infections and environmental variables. These findings may provide additional guidance to narrow the risk communication messages to metalworkers and potentially identify individuals who may benefit from the anthrax vaccine. Additionally, this model may serve as a baseline to which future cases and environmental sampling results can be added to refine the ecological niche. 

## 2. Results

**Figure 1:**
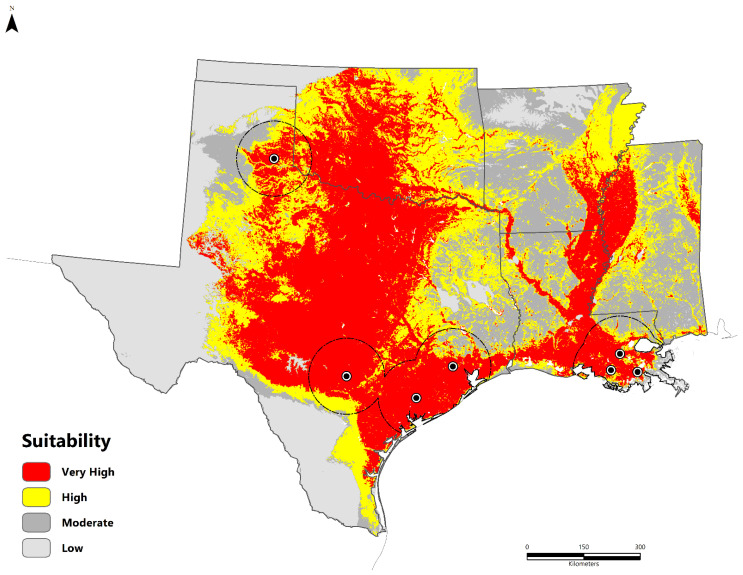
Maxent median suitability model (FC = LQP; RM = 1.5) for *B. cereus* group bacteria containing anthrax toxins. Black–white dots represent the study occurrence data (n = 7). The model calibration region (**M**) is outlined in black.

**Figure 2:**
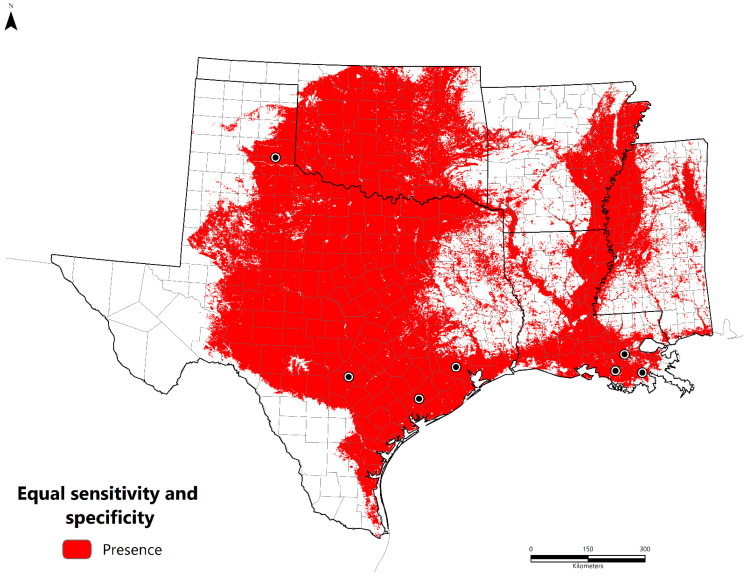
Equal training sensitivity and specificity (sensitivity = specificity) threshold (presence/absence) (value = 0.451) representing potentially suitable environments for *B. cereus*. Black–white dots represent the study occurrence data (n = 7).

We obtained *B. cereus* strains isolated from seven metalworkers between 1994 and 2020. These cases were located in Louisiana (n = 3) and Texas (n = 4). The majority were clustered along the 30th parallel north in the humid subtropical climate zone (Cfa), except the southeastern Texas panhandle case, which features a cold, semi-arid climate (Bsk). The potentially suitable areas for *B. cereus* are presented in Figure 1 as a continuous surface and binary format based on the equal training sensitivity and specificity (sensitivity = specificity) threshold value of 0.451 (Figure 2). Suitable areas are located along the Gulf Coast throughout Texas, Louisiana, and Mississippi. Inland regions include the Texas Backland Prairies, East Central Texas Plains, Edwards Plateau, Cross Timbers, Mississippi Alluvial Plain, and Central Great Plains. Areas of low suitability include the eastern temperate forests of the South-Central Plains, Ozark Highlands, Boston Mountains, Trans-Pecos Region, and Southeastern Plains. 

**Figure 3:**
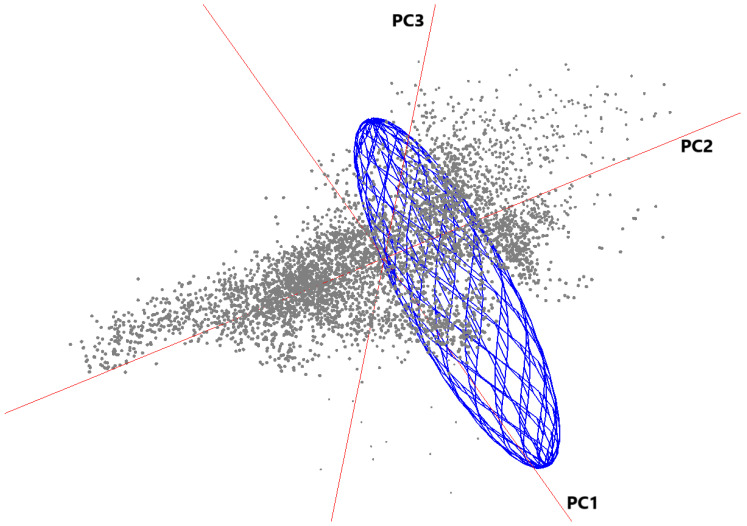
Visualization of the ecological niche of *B. cereus* based on three environmental dimensions (Set 3 PC2—x, Set 1 PC2—y, and Set 4 PC1—z) representing the principal components with the highest contribution to the Maxent model. The minimum-volume ellipsoid (blue) represents the ecological limits under which the *B. cereus* human cases were documented.

Model evaluation testing using the pROC metric determined that the *B. cereus* model yielded predictions above the null expectations with a pROC ratio of 1.85 (partial ROC = 0.922) (E = 0.05). The model variable contribution was highest for soil characteristics (cation exchange capacity, carbon content, soil pH) (PC2—27.1%; PC3—2.8%; PC1—2.7%), temperature (PC2—18.1%; PC3—11.2%; PC1—10.6%) (bio1—bio11), EVI—LST (PC1—16.5%), and precipitation (PC2—5.3%; PC1—4.6%) (bio12–bio19). In addition, the first three-axis (x, y, z) from the variables with the highest contribution to the Maxent model (Figure 3) were visualized in environmental space (E) as a minimum-volume ellipsoid (blue) calculated around the occurrences (n = 7) to illustrate the ecological limits under which the human cases were documented. Gray shading represents the available environmental background conditions. 

Additionally, we performed a separate analysis that included the Florida case location, which was excluded from the primary analysis stage. Subsequent results indicate that this location displays environmental dissimilarities compared to the Texas and Louisiana case locations. Please see the Supplemental Materials for the principal component sets variable contribution chart (Figure S1) and Maxent model projected to Florida (Figure S2) and a brief description of these findings.

## 3. Discussion

This study examined the disease ecology of *B. cereus* strains isolated from seven metalworkers between 1994 and 2020. This is the first exploratory analysis that attempted to map the ecological niche of *B. cereus* group strains with the pXO1 anthrax toxin genes in North America. Although limited to the southern United States based on the small number of observations, these exploratory findings suggest that suitable areas for the *B.* *cereus* group isolates with the anthrax toxin genes are predicted across the Gulf Coast, specifically within the limits of the humid subtropical climate zone (Cfa) and the eastern extent of the arid, steppe, cold (Bsk), and arid, steppe, hot climate types (Bsh). Soil characteristics (cation exchange capacity, carbon content, soil pH), temperature (bio1–bio11), enhanced vegetation index, land surface temperature, and precipitation were significant contributors to the model. This correlative model developed with Maxent aims to understand better the regions of potential suitability that can be further refined as more cases are identified and potentially aid in targeting educational campaigns for metalworkers to increase occupational safety measures. 

Extensive research has in the past well-characterized the environmental requirements for *B. anthracis* [13,14]; however, only Romero-Alvarez and colleagues [12] have examined the ecological niche and geographic overlap between *B. anthracis* and *B. cereus* biovar *anthracis (Bcbva)*, a member of the *B. cereus* group containing anthrax toxin genes. In addition, knowledge of the ecology and overlap of separate species in the *B. cereus* group has not been evaluated on other continents, nor has the niche been explored among this specific subset of organisms containing pXO1 toxin genes that have caused human disease. 

Previous research on the species *B. cereus*, without accounting for the presence or absence of the anthrax toxin, has demonstrated that the bacteria are widely distributed in the natural environment, mainly in soils with higher soil pH values (minimum 5–6) and water content at a minimum of 0.95 [15]. In our study, the soil pH at each location ranged from 5.9 to 7.5, with an average of 6.5. Suitability is extensive throughout soil types with greater natural fertility and more alkalinity. Luo and colleagues [16] determined that *B. cereus* and *B. thuringiensis* grew well in soils with greater nutrient availability and wetter soil conditions (at 0, −0.01 MPa) [17]. Strains of the *B. cereus* group can adapt to a wide variety of habitats, from cold to hot environments, spanning alpine and temperate soils [18,19]. Outside of soil, *B. cereus* is found in sediments, dust, clinical settings, the intestine of insects and animals, and plants [18,19,20].

Climatic influences on populations of *B. cereus* and *B. weihenstephanensis* in tropical, temperate, and alpine environments were examined by von Stetten and colleagues [19]. The distribution of isolates was greatly dependent on the annual average temperature and the magnitude of thermal fluctuation because of the prevailing climate [19]. Our results similarly determined that temperature was significant in driving suitability patterns, which has also been found to be a predictor of the ecological range of *B. anthracis* [21,22]. Regarding temperature, the Hall County case (03BB87) serves as an example of the survivability of *B. cereus*. This small county, located within the southeastern Texas panhandle, is classified as an arid, steppe, cold climate type (Bsk). The average minimum and maximum temperature in Hall County (1981–2015) ranged from −3.2 °C (26.24 °F) to 12.1 °C (53.78 °F), while the average precipitation is sparse at only 14.2 mm or 0.56 inches. Soil sampling was done at this location following the identification of the case, but no *B. cereus* group bacteria were recovered [1,3]. Further exploration of this area is needed to understand whether it is suitable or whether the area could have been contaminated with material from another location. 

Additionally, the influence of vegetation (EVI) and land-surface temperature (LST) (Set 4) contributed significantly to the Maxent model. Vegetation indices such as EVI are mathematical estimates of the quantity, structure, and conditions of vegetation extracted from spectral bands of satellite imagery [23]. Previous research has indicated that vegetation biomass is an important predictor for *B. anthracis* and *B. thuringiensis* [24,25,26]. Remotely sensed LST is a useful variable to include in ecological niche modeling because it consists of a mixture of bare soil and temperature data [27]. Furthermore, LST, along with climatic parameters such as precipitation, wind velocity, and humidity, plays an important role in various livestock-related diseases [28]. For example, previous studies have examined the relationship between spatial patterns of *B. anthracis* suitability and the influence of variables such as LST [29,30].

Studies by both Yang [21] and Blackburn [13] have previously developed broad-scale ENMs of the potential environmental suitability of *B. anthracis* in the United States. Blackburn et al. evaluated all known outbreak data [13], while Yang et al. focused on the A1.a sub-lineage [21]. The Blackburn et al. [13] model demarcated west and southwest Texas as suitable for *B. anthracis*, and Yang et al. predicted a narrower area in west-central Texas as highly suitable for A1.a. These areas are dominated by arid, steppe, cold (Bsk); and arid, steppe, hot (Bsh) climate types, which had some overlap on the western and northern borders of our predicted area and with the historic enzootic region of Texas [23]. The optimal environmental variables were a combination of temperature seasonality, precipitation, elevation, vegetation, and soil content [21]. 

Unlike both Yang’s and Blackburn’s models, suitability in this study excluded much of the South Texas Plains, northwestern High Plains, and the Trans-Pecos Region as suitable environments for *B. cereus*. Our model differs from *B. anthracis* models because most of the predicted suitable area falls within the humid subtropical climate zone with some overlap with the adjacent arid climate types. Although not directly comparable, this study has some similarities to the work of Romero-Alvarez and colleagues [12]. They determined that while displaying some overlap in their niches, some divergent characteristics between *B. anthracis* and *Bcbva* were apparent in sub-Saharan Africa. These differences were most evident in that *Bcbva*, compared to *B. anthracis*, was predicted within humid forest environments of equatorial central and west Africa.

There are several limitations to the use of ecological niche modeling to estimate the geographic scope of an organism, but foremost is the small sample size of the isolates (n = 7). However, even with the small number of locations used in this study, the application of Maxent has been documented as performing exceptionally well when comparing model predictive performance measures. In addition, it is unknown how many cases may be missed since a hemolytic and motile *Bacillus* spp. may be ruled a contaminant in a clinical laboratory or would not meet sentinel laboratory guidelines to report to the Laboratory Response Network [31]. For the confirmed cases, the estimated exposure location is only a hypothesis for six of the seven cases since only one case had a direct environmental linkage to their worksite and the infecting strain [6,32]. Our exclusion of the Florida case location was due to limited information on the potential exposure location (we only had the clinic location) [5]. Yet, we cannot preclude that these bacteria persist in central Florida. Thus, the area defined in this model may incorrectly estimate the actual geographic distribution. 

Here, the suitability model does not measure the potential prevalence or incidence and characterizes the similarities between human cases and environmental variables. While it is an important tool to measure habitat suitability, ecological niche modeling cannot predict adaptation, dispersion, or migration [33] because the true niche of *B. cereus* remains unknown in the southern United States. This model serves as a baseline on which to build as more data are collected. It may help focus future studies on understanding the occupational association of *B. cereus* group infections among metalworkers, including environmental sampling of worksites in potentially suitable areas where welding activities are performed, serosurveys to identify previous exposures, and health hazard evaluations of worksites. In addition, future research could benefit from directly comparing the potential distribution of *B. cereus* and *B. anthracis* to determine overlap and where these potential ecological transition zones are most prevalent.

## 4. Materials and Methods

### 4.1. Human Case Information

**Figure 4:**
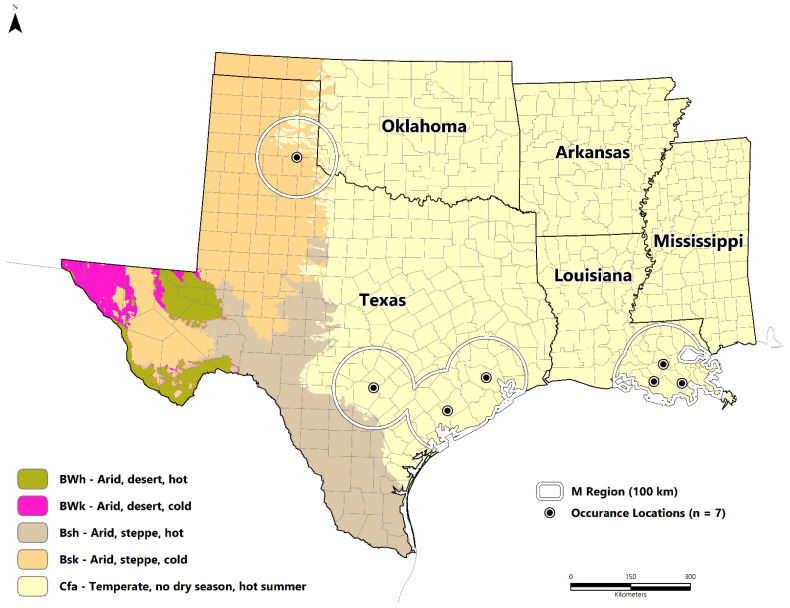
Study area and corresponding model calibration area (**M** region) compared to the Köppen-Geiger climate classification [34]. Black–white dots represent the study occurrence data (n = 7).

Seven *B. cereus* group strains with the anthrax toxin genes are included in this study; these strains were isolated from seven metalworkers between 1994 and 2020 [1,3,4,6,32] (Figure 4). Coordinates of the worksite location for each patient were used for the ecological niche model (Table 1); the worksite was used based on the hypothesis of occupational exposure, given that all were metalworkers. The Florida case was excluded from the primary analysis since the only known location was where the patient sought care. The patient did not have any known similar occupational exposure [5]. However, the Maxent model was projected to the state of Florida using the same methods described below and are briefly summarized in the Supplementary Files. 

The average patient age was 42 (standard deviation = 6.7); six were male, and one was female. Four of the cases were located in Texas and three in Louisiana. Additional clinical and epidemiologic investigation descriptions are reported elsewhere [9,32]. Spatial information for each location was manually georeferenced with Google Earth (https://earth.google.com/) (accessed on 15 December 2021). Geographic coordinates for these locations were standardized in a geographic coordinate system (WGS84) in ArcGIS Desktop 10.8.1. (ESRI. ArcGIS desktop: release 10.8.1. Environmental Systems Research Institute, Redlands, CA, USA).

### 4.2. Environmental Variables and Model Calibration Region

Gridded bioclimatic coverages were collected from the CHELSA v.2.1 (https://chelsa-climate.org/) (accessed on 15 December 2021) database (1981–2010) at a ~1 km resolution (30 arc-seconds) [35]. These bioclimatic data are derived from monthly mean, max temperature, and mean precipitation values (Table 2). We further split the bioclimatic data into two distinct sets for this study: temperature (bio1–bio11) and precipitation (bio12–bio19). Following guidance from previous studies [12,36,37,38] examining the ecological niche of *B. anthracis* and *B. cereus* biovar *anthracis*, we selected soil coverages identified as potentially important in the disease ecology of these bacteria. These variables were the cation exchange capacity (at pH 7), soil organic carbon (dg/kg), and soil pH (water). 

These were obtained at a ~1 km resolution (30 arc-seconds) from the SoilGrids (https://www.isric.org/) (accessed on 15 December 2021) database disseminated by the International Soil Reference Information Centre (ISRIC) (Table 2). Finally, we explored the potential effects of vegetation and the radiative skin temperature of land with a moderate-resolution imaging spectroradiometer (MODIS) (National Aeronautics and Space Administration (NASA)) (monthly mean) enhanced vegetation index (EVI) and mean eight-day land-surface temperature (LST) data. The EVI is a vegetation index optimized for high biomass areas and compared to the Normalized Difference Vegetation Index (NDVI), reduces issues with saturation [39]. These data were downloaded from the WorldGrids data archive [40] (~1 km) (Table 2). The accessible area (Figure 4) or **M** region served as the model calibration area [41]. Ecological niche models can be sensitive to over-parametrization and over-fitting based on the choice of the study area. Thus, the **M** region represents the hypothesized region where a species can colonize over time [12]. Our **M** region was therefore set as buffers surrounding each occurrence location (n = 7) at a 100-km extent. 

**Table 1:** Case demographics and strain information.

Case No.	County/Parish	State	Age/Sex	Date	Strain	Reference
1	Assumption	LA	42/M	1994	G9241	[2]
2	Hall	TX	39/M	2003	03BB87	[1,3]
3	Comal	TX	56/M	2003	03BB102	[1,3]
4	LaFourche	LA	47/F	2007	*B. cereus* LA 2007	[42]
5	Wharton	TX	39/M	2012	Elc2	[4]
6	St. James	LA	39/M	2020	*B. cereus* LA 2020	[6,32]
7	Harris	TX	34/M	2020	*B. cereus* TX 2020	[6,32]

**Table 2:** Environmental variables.

Variables	Units	Resolution
**Set 1**		
Annual Mean Temperature (bio1)	C°	~1 km
Mean Diurnal Range (Mean of Monthly (Max Temp–Min Temp)) (bio2)	C°	~1 km
Isothermality (bio2/bio7) (bio3)	C°	~1 km
Temperature Seasonality (Stand. Dev.) (bio4)	C°	~1 km
Max Temperature of Warmest Month (bio5)	C°	~1 km
Min Temperature of Coldest Month (bio6)	C°	~1 km
Temperature Annual Range (bio5–bio6) (bio7)	C°	~1 km
Mean Temperature of Wettest Quarter (bio8)	C°	~1 km
Mean Temperature of Driest Quarter (bio9)	C°	~1 km
Mean Temperature of Warmest Quarter (bio10)	C°	~1 km
Mean Temperature of Coldest Quarter (bio11)	C°	~1 km
**Set 2**		
Annual Precipitation (bio12)	mm	~1 km
Precipitation of Wettest Month (bio13)	mm	~1 km
Precipitation of Driest Month (bio14)	mm	~1 km
Precipitation Seasonality (Coefficient of Variation) (bio15)	mm	~1 km
Precipitation of Wettest Quarter (bio16)	mm	~1 km
Precipitation of Driest Quarter (bio17)	mm	~1 km
Precipitation of Warmest Quarter (bio18)	mm	~1 km
Precipitation of Coldest Quarter (bio19)	mm	~1 km
**Set 3**		
Cation Exchange Capacity (at ph7)	at pH 7	~1 km
Soil Organic Carbon	dg/kg	~1 km
Soil pH water	pH*10	~1 km
**Set 4**		
Enhanced Vegetation Index (EVI)	0–5.1	~1 km
Land Surface Temperature (LST)	C°	~1 km

### 4.3. Principal Component Analysis (PCA) 

Following the masking of our environmental data, a principal component analysis (PCA) was carried out in the cross-platform application Niche Analyst (NicheA) [43] (http://nichea.sourceforge.net/) (accessed on 15 December 2021) to reduce multicollinearity and dimensionality while retaining the most valuable raw information. Here, we calculated the principal component sets for the **M** region and projected these data to the whole of our study area: Texas, Louisiana, Oklahoma, Arkansas, and Mississippi. The first 3 PCs for temperature were retained (bio1–bio12) (100%) (Set 1), as were the first two for precipitation (bio12–bio19) (100%) (Set 2), all three PCs for soil (cation exchange capacity; soil organic carbon; soil pH) (100%) (Set 3), and the two sole PCs for enhanced vegetation index (EVI) and land surface temperature (LST) (100%) (Set 4).

### 4.4. Ecological Niche Modeling

The estimated ecological niche for *B. cereus* was modeled with the maximum entropy algorithm, Maxent v.3.4.4 [44]. Maxent estimates the potential geographic extent of a species by identifying the distribution with ‘maximum entropy’ subject to the constraints from the environmental conditions at the site of occurrence locations. Before modeling, we analyzed model complexity by applying a range of regularization multipliers (RM) and feature class types (FC) (mathematical transformation of predictor variables) with the R programming language package [45], ENMeval v.2.0.0 [46]. Within the ENMeval package, we specified regularization multipliers ranging from 0.5 to 2 (0.5, 1, 1.5, 2) along with three feature class types of linear (l), quadratic (q), and product (p) [47,48,49] and a ‘jackknife’ (leave-one-out) non-spatial partition, which is recommended for small data sets (i.e., <25 presence locations).

We selected LQP features since ecological gradients are frequently nonlinear [50], and the choice of LQP feature classes can result in simpler models that generate broader potential distributions [51]. The best model settings were based on the lowest (<0) corrected Akaike information criterion (AICc) (delta. AICc) [52]. Following tuning of our occurrence data (n = 7) and principal component (PC) sets (Sets 1–4) in ENMeval, we set our Maxent model to run 100 bootstrap replicates with 50% of the available data, LQP feature class type, and an RM = 1.5 (delta. AICc = 0). Additional settings specified 10,000 background points, 1000 iterations, cloglog output (inhomogeneous Poisson process (IPP)), and ‘clamping’ enabled. Due to the small number of occurrence locations, we avoided extrapolations outside the ecological range calibrated within the **M** region by enabling clamping [12]. 

Model predictive performance was determined with the partial receiver operating characteristic (pROC) metric. Partial AUC ratios, unlike the traditional AUC (area under the curve) statistic, allows for differential weighting between model omission and commission errors [53] and range from values of (partial AUC divided by random expectations) 0–2 (values of 1 = random performance). The pROC metric was calculated at a 5% error rate (E = 0.05) using the Niche Analyst (NicheA) software (http://nichea.sourceforge.net/) (accessed on 15 December 2021) [43]. Next, we converted the median suitability model to a binary output representing presence-absence with a threshold type of equal training sensitivity and specificity (sensitivity = specificity). Finally, we developed a minimum-volume ellipsoid (MVE) [54], which visualized the virtual ecological niche of *B. cereus* in environmental space (E) through the creation of a background cloud (BC) representing the first three principal components (PC) (x, y, z) with the highest overall model contribution. Moreover, the background cloud represents the available environmental conditions. The minimum-volume ellipsoid was created in the Niche Analyst software (NicheA) [43] (http://nichea.sourceforge.net/) (accessed on 15 December 2021).

## 5. Conclusions

This study estimated the ecological niche model of *B. cereus* with anthrax toxin genes based on confirmed, naturally acquired human infections in Louisiana and Texas. Environmental variables contributing to the predicted geographic distribution were the soil characteristics (cation exchange capacity, carbon content, soil pH), temperature (bio1–bio11), enhanced vegetation index (EVI), and land surface temperature (LST). Much of the suitable environments are found throughout the Gulf Coast Plain, Texas Backland Prairies, East Central Texas Plains, Edwards Plateau, Cross Timbers, Mississippi Alluvial Plain, and Central Great Plains. Despite the acknowledged limitations of this research, we believe that this study will provide a roadmap for future modeling endeavors to decipher the ecological requirements of opportunistic pathogens that pose a threat to occupational safety. Additionally, due to the severe and fatal nature of *B. cereus* with the anthrax toxin genes, our results may provide geographic information to focus future research and educational campaigns for metalworkers in the southern United States.

## Data Availability

The data presented in this study are available on request from the corresponding author. The data are not publicly available due to privacy and ethical concerns.

## Supplementary Materials

The following supporting information can be downloaded at: https://www.mdpi.com/article/10.3390/pathogens11040470/s1, Figure S1: Principal component variable contribution; Figure S2: Maxent median suitability model for *B. cereus* group bacteria containing anthrax toxins projected to Florida.
