# Supplementary material for: Ecological Niche Model of Bacillus cereus Group Isolates Containing a Homologue of the pXO1 Anthrax Toxin Genes Infecting Metalworkers in the United States
Source: Pathogens. 2022 Apr 14;11(4):470. doi: 10.3390/pathogens11040470 (PMC9027579; doi:10.3390/pathogens11040470)
Supplement: Supplementary file 1 [file pathogens-11-00470-s001.zip › pathogens-1631486-supplementary.pdf]

Supplementary Materials

SUPPORTING INFORMATION FOR: Ecological niche model of Bacillus cereus group isolates containing a homologue of the pXO1 anthrax toxin genes infecting metalworkers in the United States

Table of Contents

Figure S1: Principal component variable contributions

Figure S2: Maxent median suitability model for *B. cereus* group bacteria containing anthrax toxins projected to Florida

Brief description of findings

Figure S1: Maxent principal component variable contribution:

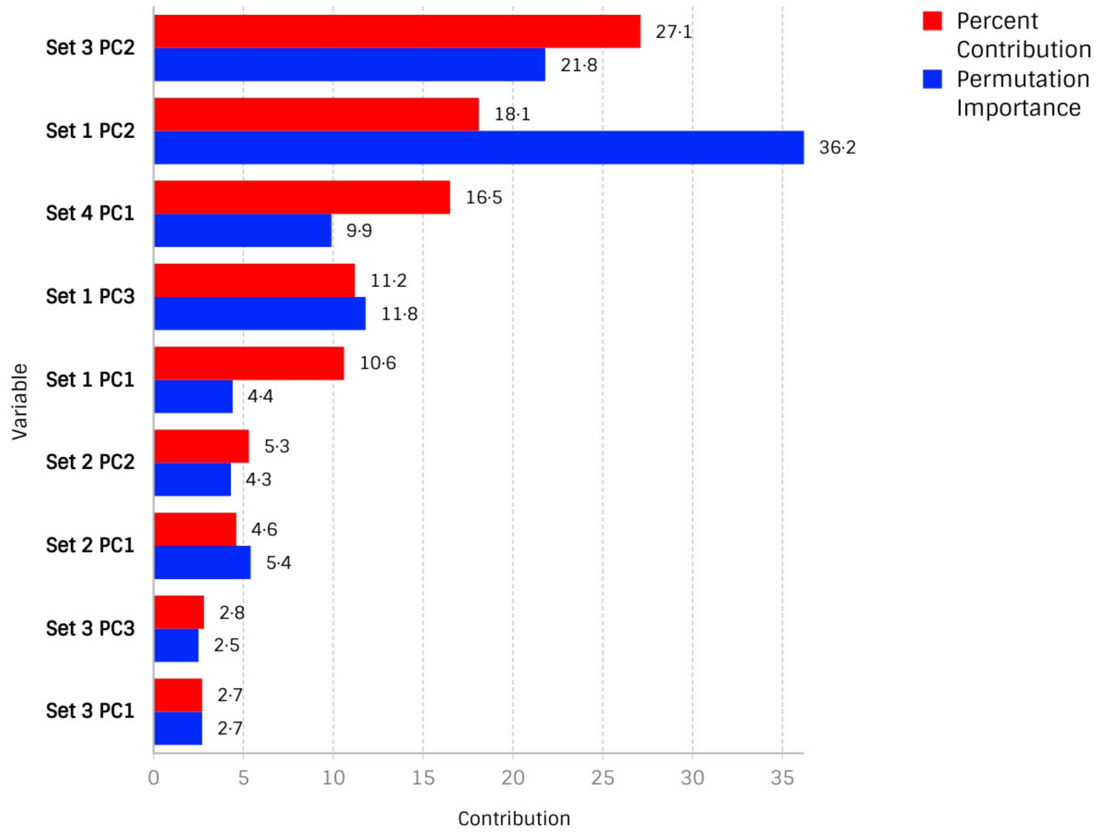

Figure S2: Model from primary analysis projected to Florida

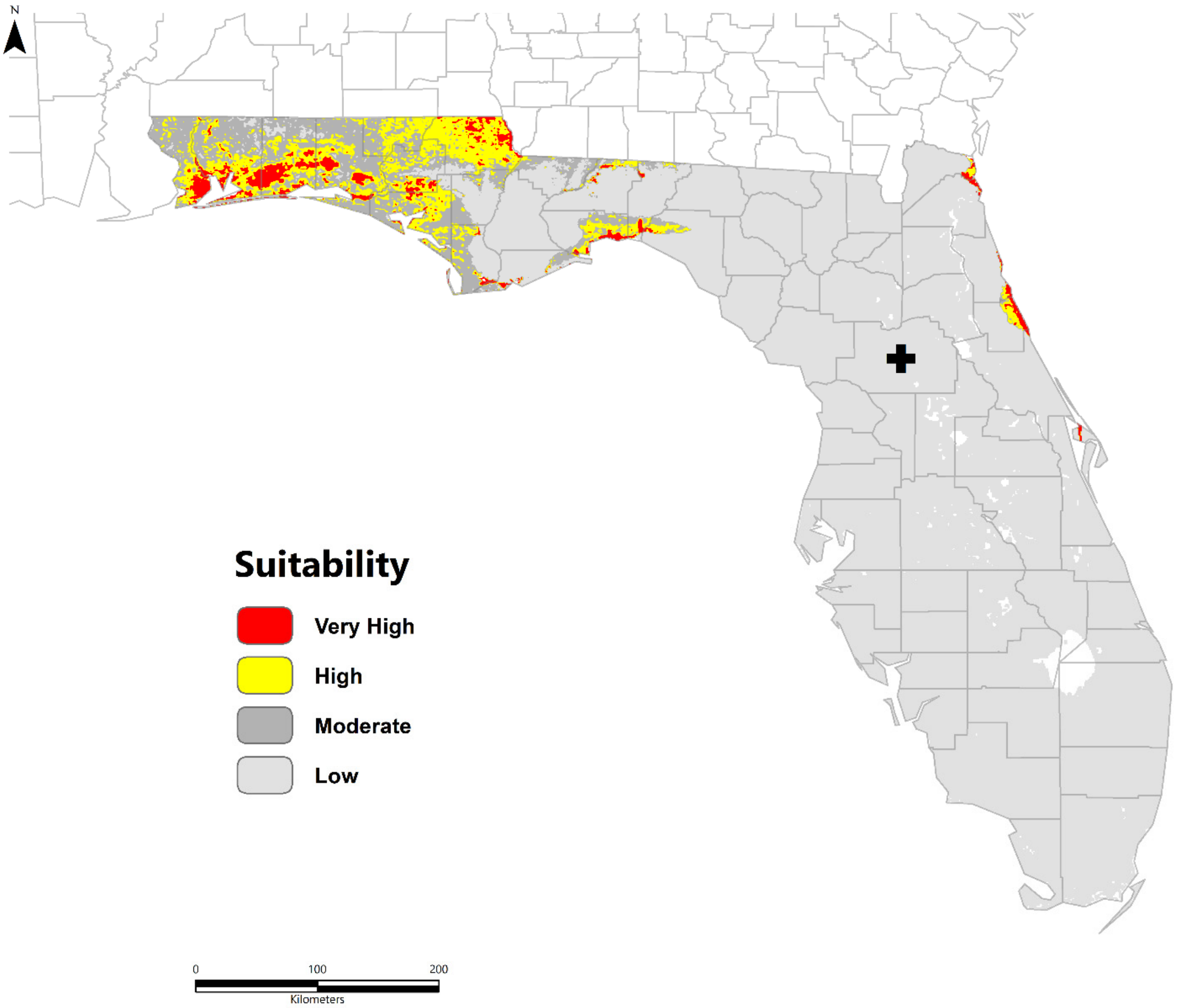

The model developed from the seven metalworker cases was projected to the state of Florida, with the location where the Florida patient sought care. Additional information was not available for potential location(s) of exposure, although it is known that the patient did not travel to Texas or Louisiana. Although our estimated model does not indicate suitable ecological conditions for a local exposure in central Florida, we cannot disqualify that exposure occurred elsewhere. More research is needed to understand if Florida could contain suitable ecological conditions for survival of *B. cereus* group bacteria with anthrax toxins.
